# Molecular Genetics of Frontotemporal Dementia Elucidated by *Drosophila* Models—Defects in Endosomal–Lysosomal Pathway

**DOI:** 10.3390/ijms19061714

**Published:** 2018-06-09

**Authors:** Sarah E. Vandal, Xiaoyue Zheng, S. Tariq Ahmad

**Affiliations:** Department of Biology, Colby College, 5720 Mayflower Hill, Waterville, ME 04901, USA; sevandal@colby.edu (S.E.V.); xzheng20@colby.edu (X.Z.)

**Keywords:** frontotemporal dementia, endosomal-lysosomal pathway, ESCRT, CHMP2B, CHMP2B^intron5^, animal models, *Drosophila*

## Abstract

Frontotemporal dementia (FTD) is the second most common senile neurodegenerative disease. FTD is a heterogeneous disease that can be classified into several subtypes. A mutation in *CHMP2B* locus (*CHMP2B^intron5^*), which encodes a component of endosomal sorting complex required for transport-III (ESCRT-III), is associated with a rare hereditary subtype of FTD linked to chromosome 3 (FTD-3). ESCRT is involved in critical cellular processes such as multivesicular body (MVB) formation during endosomal–lysosomal pathway and autophagy. ESCRT mutants causes diverse physiological defects primarily due to accumulation of endosomes and defective MVBs resulting in misregulation of signaling pathways. Charged multivesicular body protein 2B (CHMP2B) is important for neuronal physiology which especially rely on precise regulation of protein homeostasis due to their post-mitotic status. *Drosophila* has proven to be an excellent model for charaterization of mechanistic underpinning of neurodegenerative disorders including FTD. In this review, current understanding of various FTD-related mutations is discussed with a focus on *Drosophila* models of CHMP2B^intron5^-associated FTD.

## 1. Introduction

Frontotemporal dementia (FTD) is the second most common form of dementia, and the second most common neurodegenerative disease in individuals under the age of 65 [1]. It is characterized by progressive impairment of language and executive functioning, as well as behavioral modifications, due to degeneration of the frontal and temporal lobes of the brain [2,3]. FTD is a heterogeneous disease because it can result from a multitude of genetic mutations [4]. FTD also shares similarities with various other neurodegenerative disorders, such as progressive supranuclear palsy (PSP) syndromes, corticobasal degeneration (CBD), and argyrophilic disease (AGD) and amyotrophic lateral sclerosis (ALS), suggesting common neuropathological mechanisms to neurodegeneration [2,3]. FTD is divided into different subtypes which encompass a range of molecular and behavioral deficits. One subtype is the behavioral variant of FTD (bvFTD), which is typically classified by gradual changes in behavior, cognition, or both. Another subtype is primary progressive aphasia (PPA), which is a language disorder that results from temporal lobe atrophy. This disorder is further divided into additional subgroups which are categorized based on a spectrum of the areas of language affected: logopenic variant PPA (lvPPA), nonfluent variant PPA (nfvPPA), and semantic dementia (SD) [2,3,4].

An additional subtype of FTD, called frontotemporal dementia linked to chromosome 3 (FTD-3), is dominantly inherited and is characterized by selective cortical neuron degeneration. FTD-3 patients display cognitive impairment, social behavior deficits, and dystonia, which typically appear between the ages of 46 and 67 [1]. For this reason, FTD-3 is classified under the FTD subtype of frontotemporal lobar dementia [5]. One of the genetic defects associated with FTD-3 is a mutation in the *CHMP2B* gene; a single nucleotide mutation (G to C) at the splice site of CHMP2B exon 6. This variation establishes two abnormal transcripts, *CHMP2B^intron5^* and *CHMP2B^Δ10^*, both of which encode for proteins with a defective carboxyl terminus [5,6,7].

**Figure 1:**
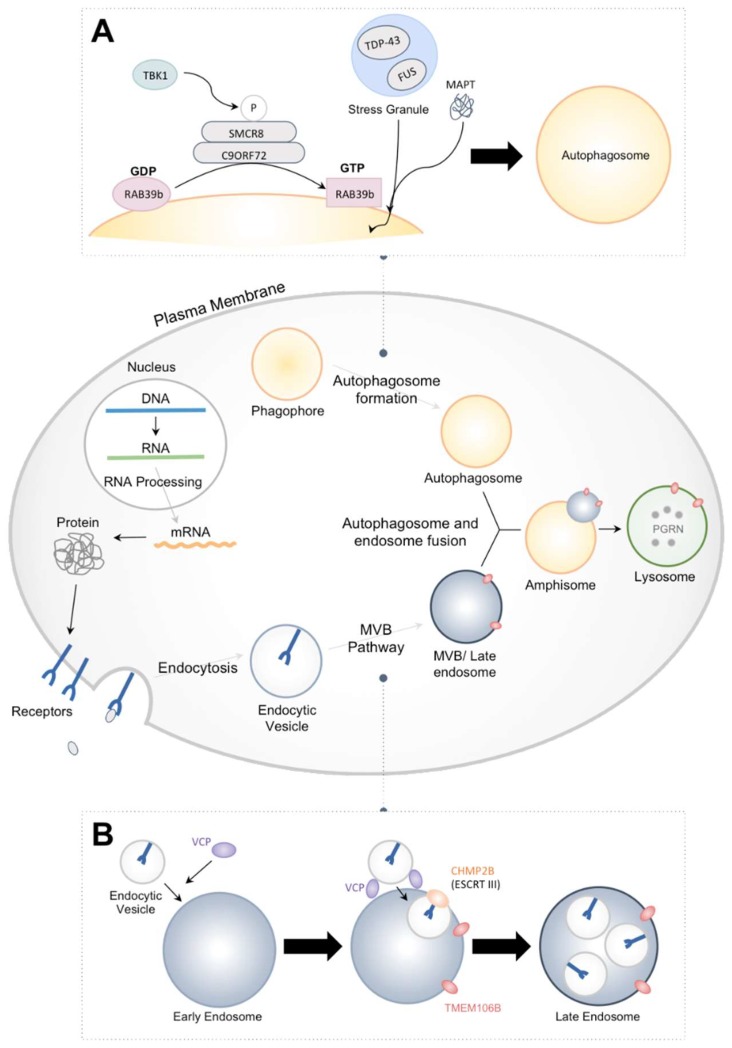
Role of proteins associated with frontotemporal dementia (FTD) in endosomal–lysosomal and autophagy pathway. FTD associated proteins, highlighted in yellow, are involved in both autophagy and endosomal–lysosomal pathways. (**A**) Phagophores in the cytoplasm undergo autophagosome formation with the assistance of C9ORF72, TANK binding kinase 1 (TBK1), microtubule-associated protein tau (MAPT), transactive DNA-binding protein (TDP-43), and fused in sarcoma (FUS) proteins, as well as various other protein complexes not included in the figure above. (**B**) Endocytic vesicles bearing receptor cargo transition through maturation stages to form MVBs and eventually fuse with the lysosomes. ESCRT complexes and additional proteins, including valosin containing protein (VCP) and transmembrane protein 106B (TMEM106B), contribute in the development of the endosome and MVB pathway in the endosomal–lysosomal pathway.

This review will focus on the most recent findings concerning FTD-associated mutations, particularly on the *CHMP2B^intron5^* mutation and its effect on misregulation of receptor-mediated signaling due to perturbation of the endosome–lysosome pathway and autophagosome–lysosome pathway (Figure 1). *Drosophila* and mouse models have made great strides in discovering the underlying mechanisms of the CHMP2B*^intron5^*-mediated phenotypes.

## 2. Mutations Associated with FTD

Aside from the mutant *CHMP2B^intron5^*, numerous mutations in many other loci are associated with FTD (Table 1; for review of molecular genetics of FTD, see Rainero et al. [8]). In this section we have provided a brief overview of the mutations associated with FTD. We have also briefly included the major conclusions obtained from the *Drosophila* model of the mutations. The most common mutations occur in the genes *C9ORF72* (chromosome 9 open reading frame 72), *PGRN* (progranulin), and *MAPT* (Microtubule associated protein tau). Alterations in the *C9ORF72* locus is the most prevalent cause of FTD (25% of all FTD cases) [1]. The GGGGCC hexanucleotide repeat expansion located in intron 1 of this gene is the source of pathogenicity [9,10,11]. While 2 to 24 repeats are typical in normal individuals, hundreds to thousands of these repeats are present in the affected individuals. The expanded repeats cause abnormal translation of *C9ORF72* transcript resulting in toxic dipeptide repeats (DPRs) through a mechanism known as repeat associated non-ATG translation (RAN) [9,12]. Comprehensive analysis by Mizielinska et al., using a *Drosophila* model, has shown that a toxic DPR secondary structure is not necessary to cause neurodegeneration in adult flies; instead, the aberrant translation of RNA is sufficient [13]. DPRs are particularly important in enhancing or suppressing nuclear import or export of proteins. Additionally, C9ORF72, along with SMCR8 and WDR41, are associated with autophagosome formation, and loss of C9ORF72 is associated with defects in autophagosome formation in cell culture and mouse models (Figure 1A) [14,15].

Another locus associated with FTD is *GRN* which encodes for PGRN, a growth factor involved in multiple pathways including related to inflammation [16,17]. FTD associated with nonsense, splice-site, or frameshift variants of *GRN* is inherited in an autosomal dominant mode [5,18,19]. PGRN has a fundamental role in lysosome functioning and in tissue development and inflammation [1,18]. The reduction of PGRN levels from these mutations have been known to result in haploinsufficiency, a major cause of FTD [5,18]. Interestingly, there are no obvious homologs of *GRN* in *Drosophila* [20].

The *MAPT* gene encodes for the protein Tau which is responsible for stabilizing and assembling microtubules and also regulating vesicle transport regulated by kinesin [1,18,21]. There are currently 54 identified pathogenic *MAPT* mutations associated with the onset of neurodegenerative diseases, including FTD. Presence of a mutation in *MAPT* generally prevents binding between Tau and Tubulin, subsequently causing hyperphosphorylated Tau accumulation—a common neurodegenerative disease precursor [1]. *Drosophila* models of MAPT have successfully identified various Tau-induced neurodegeneration modifiers, including eight Tau toxicity suppressors and 16 enhancers [22].

*TMEM106B* serves as a genetic modifier most notably in patients carrying a mutation in *C9ORF72* and *PGRN*. While the S185 isoform of *TMEM106B* provides some protection against neurodegeneration, the isoform T185 raises risk [23]. Overexpression of *TMEM106B* results in the disruption of lysosome functioning as well as impairment of protein trafficking regulation [18]. Research presented by Jun et al. and Rostgard et al. propose a potential link between *TMEM106B* and autophagy, and more specifically the ESCRT pathway and *CHMP2B* [23,24]. Overall, the *Drosophila* models have been particularly useful in identifying the pathogenicity of the mutated proteins as well as in drug targeting investigation [19].

Mutations in *CHMP2B*, (chromatin modifying protein 2B), *FUS* (fused in sarcoma), *TARDBP* (Transactive DNA-binding protein), *VCP* (Valosin containing protein), *TBK-1* (TANK-binding kinase 1) have also been associated with FTD but are much rarer in incidence (Table 1). Together, mutations in these genes account for fewer than 3% of FTD cases. Interestingly, mutations in *FUS*, *TARDBP*, *VCP*, and *TBK-1* are also associated with ALS [8,19]. The *TARDP* locus codes for TDP-43 which is involved in multiple RNA-related gene expression events including transcription, splicing, transport (including transport of RNA granules to dendrites [25]), and translation [26]. Identification of TDP-43 variants as a major component in inclusions associated with neurodegenerative diseases including ALS and FTD led to increased interest to characterize its function and role in neuroanal pathology [27,28,29]. The *Drosophila* models of TDP-43–associated FTD/ALS were able to recreate most aspects of human proteinopathies. Further, these models also provided additional levels of characterization, for instance, neurodegeneration was not always dependent on formation of inclusions [30,31].

The *FUS* locus encodes for an RNA binding protein that is a component of stress granules, with the primary function of DNA repair and regulation of RNA splicing [26]. FUS share remarkable structural and functional similarity with TDP-43. Mutations in *FUS* result in cytoplasmic aggregate formation as well as stabilization of stress granules, restricting the disassembly of the granules [18,26]. *Drosophila* model of mutant FUS-associated toxicity recreate ALS/FTD pathology e.g., aggregation of ubiquitinated proteins, neurodegeneration, behavioral deficits in locomotion, and early mortality [32].

*VCP* encodes for an ATPase that has a wide range of cellular functions including degradation of endoplasmic-reticulum-associated proteins, delivery and unfolding of ubiquitinated proteins, and regulation of endosome morphology [2,18]. *VCP* mutations have also been found to inhibit autophagy-mediated turnover of stress granules [18]. *Drosophila* homolog of *VCP* plays a role in dendritic pruning by misregulating RNA-binding proteins including TDP-43 [33]. Interestingly, ectopic expression of disease-associated mutant VCP in *Drosophila* cause neurodegeneration due to mislocalization of TDP-43 to cytoplasm [34]. Further, VCP was also shown to genetically interact with FUS in *Drosophila*. Taken together, *Drosophila* models employing ectopic overexpression of disease-associated mutations and analysis of loss of function of *Drosophila* homologs of disease-associated loci i.e., *cabeza* (FUS), *tbph* (TDP-43), and *ter94* (VCP) have shown genetic interaction between FUS, TDP-43, and VCP. Such studies further highlight the utility of *Drosophila* models to identify and characterize gene networks that contribute in complex neurological diseases [32,35,36].

Recently, haploinsufficiency of TBK1 locus was associated with FTD and ALS [37]. Reduction in TBK1 levels due to mutations show deficits in autophagy pathway [38,39]. TBK1 influences autophagy pathway by regulating the activity/binding of p62 (nucleoporin 62) and OPTN (optineurin)—autophagy adaptor proteins. Consequently, P62 and OPTN also engage in the pathology of the ALS/FTD spectrum [39,40]. Interestingly, there is an interaction between TBK1 and OPTN with the Ras-related protein Rab8 which also interacts with the C9ORF72 complex [14]. Rab8-mediated signaling is also misregulated in a *Drosophila* model of FTD associated with mutant CHMP2B (next section of this review) [41]. Overall, *Drosophila* models of FTD-associated mutations have significantly contributed in identification, characterization, or both, of mechanistic details about the incidence and progression of the disorder.

## 3. FTD Associated with Mutant CHMP2B

### 3.1. Structure and Function of CHMP2B

CHMP2B is a subunit of the multi-protein ESCRT-III complex (Figure 1). The ESCRTs are composed of multiple highly conserved heteromeric protein sub-complexes, namely ESCRT-0 (hepatocyte growth factor-regulated substrate (HRS), signal-transducing adaptor molecule (STAM1-2)), ESCRT-I (tumor susceptibility gene (TSG101), vacuolar protein sorting 28 (Vps28), Vps37, MVB12, ubiquitin-associated protein UBAP), ESCRT-II ((ELL-associated protein 20 (EAP20), EAP30, EAP45), ESCRT-III (CHMP1A-B, CHMP2A-B, CHMP3, CHMP4, CHMP5, CHMP6, CHMP7), and Vps4 [42]. Although human isoforms are of ESCRTs are listed above in parenthesis, all ESCRT components are often interchangeably referred by nomenclature from yeast. Seminal work in the *Saccharomyces* model system have led to discovery and mostly contributed to the characterization of the ESCRT pathway [43]. The ESCRT machinery is involved in membrane remodeling and scission during various critical cellular processes, such as multivesicular body (MVB) formation in endosomal–lysosomal pathway [44], retrovirus budding [45], and cytokinesis [46]. Recently, ESCRT was also shown to mediate other important cellular activities for example repair and homeostasis of plasma and nuclear membranes, protein secretion, and dendritic pruning (For review, see Christ et al., 2017) [47]. This section focuses on ESCRT-mediated endosomal sorting and macroautophagy, which are two processes so far shown to be primarily affected by CHMP2B mutation. In addition, the role of ESCRT in the nervous system is discussed.

The MVB pathway is initiated by the ESCRT-0 complex. By binding both phosphatidylinositol 3-phosphate (PtdIns3P) and ubiquitin, ESCRT-0 binds to the endosomal membrane and clusters the ubiquinated cargo protein. ESCRT-0 also recruits ESCRT-I, which in turn recruits ESCRT-II. The two complexes induce membrane budding and confine the cargo. ESCRT-II then initiates ESCRT-III assembly by binding and activating CHMP6 (Vps20). The active CHMP6 instigates CHMP4 (Snf7) homo-oligomerization, which is capped by CHMP3 (Vps24). CHMP3 finally recruits CHMP2 (A and B) and thus completes the assembly of ESCRT-III [4,42,48,49,50,51]. In addition, interactions between Vps28 of ESCRT-I and CHMP6 of ESCRT-III were observed, suggesting existence of direct connections between ESCRT-I and III [52,53]. Other accessory proteins, such as ALG-2-interacting-protein-X (ALIX), also contribute to facilitate the recruitment of ESCRT-III [48]. CHMP2, together with other ESCRT-III subunits, subsequently recruits AAA ATPase Vps4 by binding the MIT domain on the N-terminus. The recruited Vps4 assembles to a dodecomer and binds its cofactor Vta1 to form the Vps4-Vta1 complex [42,51]. Vps4 provides the ATPase input in the membrane scission, and it also rapidly depolymerizes ESCRT-III filaments, a process essential to the recycling of ESCRT-III subunits [54,55,56].

CHMP2B is a highly conserved protein of 213 amino acids that contains coiled-coil, Snf-7, and acidic C-terminus domains [7]. The coiled-coil domain functions as a molecular spacer and mediates membrane tethering of vesicles [57]. The acidic C-terminus domain contains a microtubule interacting and transport (MIT)-interacting motif (MIM), which can form an amphipathic helix that binds the Vps4 MIT domain [58,59]. The recruited Vps4 catalyzes the disassembly of ESCRT-III [60]. In the cytosol, CHMP2B and other ESCRT-III subunits exist in an autoinhibition monomer. When autoinhibition is released, the subunits can polymerize into circular or helical filaments on the cellular membrane [51,61,62,63,64,65]. In particular, CHMP2B plays a structural role in membrane scaffolding directly. Overexpression of CHMP2B can form full-length polymers of the protein and create membrane protrusions [60].

Cargos sorted by the ESCRT machinery are mostly ubiquinated transmembrane proteins i.e., cell-surface receptors. The ESCRT machinery therefore plays a role in the degradation and recycling of receptors, and thus regulates the strength and timing of the signaling pathways. Receptor tyrosine kinases (RTKs) such as epidermal growth factor receptor (EGFR) and G-protein coupled receptors (GPCRs) are the two best studied cases [66,67,68,69]; other receptors regulated by ESCRTs include integrin, cytokine receptors, Toll-like receptors, and Notch receptors (For review, see Szymanska et al., 2018; receptors identified using animal model of CHMP2B mutations are discussed in next section) [70,71,72,73,74]. Several studies in *Drosophila* using mutant ESCRT subunits have shown a role of ESCRTs in tumorigenesis because of their ability to sequester Notch and JAK-STAT pathways [75,76,77,78]. These studies have raised an intriguing possibility of utilizing ESCRTs for tumor-suppression strategies.

In addition to endocytosis, ESCRTs also plays a role in macroautophagy (often referred to as autophagy). Autophagy is a conserved process of degradation of cytosolic proteins and organelles induced by various stresses, such as starvation. The initial step of autophagy is the formation of the cargo-containing double-membrane autophagosomes. The autophagosomes then fuse with lysosomes to form autolysosomes, where the cargos are degraded (Figure 1B). In this process, ESCRT machinery and endosomes are involved through the formation of amphisome, the result of autophagosomes and endosomes fusion (For review, see Lefebvre et al., 2018) [79,80]. In addition to the amphisome formation, ESCRTs also plays a role in autophagosome maturation by interacting with SNAREs. In particular, CHMP2B was shown to interact with Syntaxin 13 [81]. Recent studies also showed other interactions between specific ESCRT proteins and autophagy related proteins, suggesting a close connection between the two pathways (For review, see Lefebvre et al., 2018) [80].

ESCRT functions are crucial in the nervous system. During embryogenesis of the central nervous system, ESCRT controls the survival of neural progenitors. In the later stages of development, ESCRT regulates the outgrowth and pruning. In the mature nervous system, ESCRTs plays a role in regulating synaptic transmissions. (For review, see Sadoul et al., 2018) [82]. In particular, CHMP2B is involved in regulating synaptic plasticity in dendrite spines [6,83].

### 3.2. Defects Caused by CHMP2B^intron5^

At the molecular level, the G-C mutation in the *CHMP2B* transcript causes the inclusion of the 201-bp intron 5, which results in addition of a valine residue followed by a stop codon. Hence, CHMP2B^intron5^ features a C-terminus truncation, with the last 36 amino acids replaced by a valine [7]. The truncated part of C-terminal region contains the MIM domain, which is responsible for Vps4 recruitment, so CHMP2B^intron5^ cannot interact with Vps4 [60]. As a result, the membrane scission function of ESCRT is impaired, and therefore the MVB pathway and the autophagy pathway are disrupted. Also, since the C-terminal domain is essential to the autoinhibition of CHMP2B, the mutant protein is constitutively active, resulting in polymerization that deform the membrane [60].

Disruption of the endosomal–lysosomal pathway by CHMP2B^intron5^ primarily causes misregulation of receptor turnover. The impaired degradation of receptors results in upregulation of receptor-mediated signaling pathways. Because of the relative ease of ectopic expression of genes with control over spatio-temporal attributes and the capacity to conduct genetic screens, studies using the *Drosophila* model of CHMP2B^intron5^ have led to the identification of multiple misregulated receptor-mediated signaling. Ectopic expression of CHMP2B^intron5^ primarily during photoreceptor cell diffrentiation in the *Drosophila* eye imaginal disc resulted in excessive melanization in the eyes due to upregulation of Toll receptor-mediated signaling, a conserved pathway of innate immune response [84]. Additionally, ectopic expression of CHMP2B^intron5^ during eye tissue specification during larval stages caused tumorigenic deformities in the adults eyes due to upregulation of Notch pathway—a fundamental cellular interaction pathway involved in cell fate determination and differentiation during development [85]. Furthermore, Transforming Growth Factor beta (TGF-β) and c-Jun N-terminal kinases (JNK), Rab8 signaling are upregulated when CHMP2B^intron5^ is expressed in *Drosophila* neuromuscular junctions (NMJ). The resulting synaptic overgrowth at the NMJ synapse is a distinct feature of neurodegeneration [41]. Recently, the pro-apoptotic protein POSH/SH3RF1 was shown to mediate the JNK and NF-κB dependent apoptosis in CHMP2B^intron5^–mediated toxicity in *Drosophila* NMJ [86].

ESCRT machinery also plays a role in autophagosome formation. Therefore, CHMP2B^intron5^ also induces defects in autophagy. Recent genetic interaction evidence suggests that the mutant protein inhibits phagophore maturation through Syntaxin 13 [81]. Ectopic expression of CHMP2B^intron5^ was shown to cause accumulation of autophagosomes, which contained aggregates of ubiquinated proteins [87,88].

Although the *Drosophila* model provides major advantages in identification and characterization of genetic modifiers and pathological mechanisms of mutations associated with FTD, alternative models e.g., knock out and trangenic mice, primary neuronal culture, and neuronal culture derived from induced pluripotent stem cell (iPSC) and Human embryonic stem cell (hESC) lines have significantly contributed in understanding to further progress discoveries in these areas [19]. When expressed in mouse neurons, CHMP2B^intron5^ was shown to cause behavioral and histological features of FTD and ALS. In particular, CHMP2B^intron5^ mouse exhibited behavioral deficits as well as progressive loss of motor activities [89]. On the cellular level, CHMP2B^intron5^ effects dendrites by inducing decrease in spine density and reduction in mushroom spine morphology [6]. In mouse models, in addition to ubiquinated protein aggregation, CHMP2B^intron5^ also induces gliosis and axonal swelling [90]. Recently, using cortical mouse neurons Transmembrane protein 106B (TMEM106B) was identified as a modifier of CHMP2B^intron5^ toxicity through the autophagy pathway [23]. This research identified an association between TMEM106B and CHMP2B, particularly the localization of TMEM106B variants in Rab5 and Rab7 positive endosomes, suggesting possible engagement of ESCRT pathways [23].

CHMP2B^intron5^ transfected cell lines showed reduced fusion of endosomes with lysosomes and delayed degradation of Epidermal Growth Factor (EGF) and EGFR [91]. Human embryonic stem cell (hESC)-derived postmitotic neurons were used to show the functions of ESCRT-III subunit Snf7-1 and Snf7-2, which play key roles in the neurotoxicity of CHMP2B^intron5^ [92]. A human-induced pluripotent stem cell (iPSC) line with the CHMP2B^intron5^ mutation was also generated recently using the CRISPR-Cas9 system [93]. The forebrain-type cortical type neuron differentiated from the iPSC line exhibited defects in endosome and mitochondria, increased oxidative stress, as well as perturbed iron homeostasis [94].

Another pathology caused by CHMP2B^intron5^ in mouse models is a lysosome storage disorder of progressive neuronal autofluorescent aggregate formation [95]. In addition to defects in protein trafficking and autophagy, there is also evidence that CHMP2B^intron5^ causes defects in the miRNA pathway. In particular, it was shown that CHMP2B^intron5^ reduces the level of miR-124, which leads to decrease in AMPA receptor (AMPAR) abundance [96].

## 4. Future Perspectives

Since the first systematic characterization of CHMP2B^intron5^ in 2005, there have been great strides in the understanding of the function of CHMP2B and its role in pathological mechanisms associated with FTD [39]. However, there is still much that can be discovered on this topic. One area that has seen increased interest is in identification of receptor-mediated signaling pathways and their modifiers affected by CHMP2B^intron5^. Using the *Drosophila* model, genetic screens has identified signaling pathways and their modifiers such as Toll, Notch, Syntaxin 13, Rab8, JNK, TGF-β pathways [21,41,81,84,86]. Although preliminary information is known about the function of these modifiers in their respective pathways, further investigation would provide an even better understanding of their role in these pathways and in the development of FTD in general.

Additionally, *Drosophila* has been used for a number of FTD-related mutations, there has still yet to be a model of the mutations *PGRN*, *TBK1*, and *THEM106B*. Characterization of these mutations using the *Drosophila* model would provide insight into how these mutations affect the typical functioning of the pathways they are involved in, and thus their role in FTD pathology.

With increasing advances in biotechnology, future work in in identification and characterization of molecular-genetic markers of FTD is warranted. Next-Generation Sequencing (NGS) has made significant contributions to phenotypic dissections and molecular genetics of neurological diseases [97]. Particularly, RNA sequencing (RNA-seq) analysis was utilized in the study of FTD3 to confirm defects in endosome and mitochondria and to reveal the imbalance of iron homeostasis [94]. This method is particularly beneficial as it is able to detect transcripts regardless of whether or not there is an existing characterized genomic sequence. Also, it results in very minimal background allowing for a large dynamic expression range for transcript detection [98]. Human iPSC-derived neuronal culture systems also offers exciting possibilities for longitudinal analysis for molecular pathology and drug development of FTD and other neurodegenerative disorders. Animal models of FTD will continue to contribute in improving overall understanding of the disease and development of effective therapeutic treatment of FTD. Furthermore, studies with these models will provide insights into mechanisms responsible for the same mutations manifesting into different aspects of the FTD-ALS spectrum.

## 5. Conclusions

In this review we have identified the recent findings of FTD related mutations with emphasis on CHMP2B^intron5^, in order to understand their pathological neurodegeneration mechanisms. Although rare in FTD incidence, CHMP2B^intron5^ mutation causes various molecular and cellular deficits in the endolysosomal and autophagosome–lysosome pathways. *Drosophila* have provided an inexpensive, genetically tractable, and high throughput animal model into understanding the defects caused by CHMP2B^intron5^ mutation as well as other FTD associated mutations.

## Figures and Tables

**Table 1:** Classification of FTD-associated loci.

Gene Name	C9ORF72	MAPT	CHMP2B	FUS	VCP	TARDBP (TDP-43)	PGRN	TBK1	TMEM106B
**Full Name**	Chromosome 9 open reading frame 72	Microtubule—associated protein tau	Chromatin modifying protein 2B	Fused in sarcoma	Valosin containing protein	Transactive DNA-binding protein	Progranulin	TANK-binding kinase 1	Transmembrane protein 106B
**Location**	9p21.2	17q21.32	3p11.2	16q11.22	9p13.3	1p36.22	17q21.32	12q14.2	7q21.3
**Incidence Rate**	25%	Familial: 10–20% Sporadic: 0–3%	Rare	Rare	1.6%	Rare (<20 cases)	Familial: 5–20% Sporadic: 1–5%	1.1%	Unknown
**Normal Function**	role in autophagy part of a complex that serves as a GDP-GTP exchange factor for RAB8a and RAB39b	stabilization of microtubules promotion of microtubule tubulin binding	necessary for transport in endosomal sorting complex (ESCRT-III)	involved in RNA processing and DNA repair	transcriptional activation apoptosis protein degradation membrane fusion	RNA metabolism regulator	growth factor for neurons and other cells	engages in various cell signaling pathways, including immune response, cell proliferation and growth	regulation of protein trafficking, lysosome size, and lysosome motility
**Deficits Caused**	lysosome accumulation abnormal microglia immune response	disruption of normal tau binding to tubulin causing hyperphosphorylated tau build up	build up of vesicular structures and autophagosome neuronal cell loss, and dendritic retraction	reduction of dendrite arborization in spinal neurons	disruption of autophagy protein degradation by ubiquitin-proteasome	increases B-cell lymphoma 2 (Bcl-2) mediated apoptosis caused by improper regulation of calcium signaling	low PGRN levels and accumulation of truncated granulin domain cause haploinsufficiency	unknown method of pathogenicity	causes lysosome enlargement impaired endo-lysosome degradation
**Drosophila Model**	Yes ^13^	Yes ^22^	Yes ^41, 81, 84–86^	Yes ^32^	Yes ^34^	Yes ^32^	No	No	No
**Drosophila Homolog**	None	tau/ CG45110	CG4618	*caz*	*ter94*	*TBPH*	N/A	N/A	N/A
